# Learning Organization Profile of Educational Hospitals in Iran: Practice of Organizational Interlocking Systems

**DOI:** 10.5539/gjhs.v7n5p51

**Published:** 2015-02-24

**Authors:** Rafat Mohebbifar, Hassan Jahani Hashemi, Roya Rajaee, Marziye Najafi, Mahbobeh GH Etedal

**Affiliations:** 1 School of Health, Qazvin University of Medical Sciences, Qazvin, Iran; 2 School of Management, Shahid Beheshti University of Medical Science, Tehran, Iran; 3 Health Care Administration in Tehran university of Medical Science, Emam Khomeini Hospital, Tehran, Iran; 4 Tehran University Health Network, Shahre Rey, Iran

**Keywords:** learning organization profile, learning organization, organizational learning, organizational interlocking systems

## Abstract

**Background:**

Organizational learning has been identified as necessary for different organizations to improve their performance in the changing and competitive environment.

**Purpose:**

The main purpose of this research was to specify the learning organization profile of educational and health centers of Tehran and Qazvin Universities of Medical Sciences in Iran.

**Methodology:**

The present research was conducted using a cross-sectional method in the academic year of 2013-2014. A staff of 530 from educational hospitals subordinated to Tehran and Qazvin universities of medical sciences participated in the research. The participants were selected using stratified random sampling. That is to say, a random sample of a proportionate size was selected from each hospital. The instrument for data collection was a Likert-scale questionnaire involving 50 items. The statistical techniques of ANOVA, t-test, Chi-square, correlation coefficients (Pearson and Spearman), and regression were utilized to analyze the data. All of them were performed using the Statistical Package for Social Sciences (SPSS) 16.0 for windows.

**Result:**

The results indicated that 449 of participants (84.7%) had a B.S. degree and 78 of them (14.7%) had an M.S. or a Ph.D. degree. Among the fivefold dimensions of “Learning Organization” model (Learning, Organization, People, Knowledge, and Technology) in comparison of the two universities, the “people” dimension was the highest-rated dimension with the mean rating of 25.71±8.36 and the “learning” dimension was the lowest-rated dimension with the mean of 25.35±8.04. Comparison between the two universities yielded the result that educational hospitals in Tehran University of medical sciences with the rating of 126.56 had a more complete profile than that of educational hospitals in Qazvin university of medical sciences with the rating of 122.23.

**Conclusion:**

The hospitals of the two above-mentioned universities were, to a great extent, far from the characteristics of Learning Organization. In light of the massive mission of these centers to maintain and improve the community health and to train the skilled labor force, the centers should embark on updating the data and institutionalizing learning. Furthermore, to modify staff’s behavior and performance and to achieve their goals, they should accentuate the importance of acquiring, creating, and transferring knowledge.

## 1. Introduction

We live in a world of disruptive change. In our disruptive world, an organization’s capacity to learn, apply and spread new insight has been touted as the fundamental strategic capability and leading source of competitive advantage. Organizational learning is fundamental for improving performance within a rapidly changing and competitive business environment. Some researchers argue in general that organizational learning is conducive to companies performing well in the competitive environment of today’s business world. Organizational learning can be defined as the development of new knowledge that has the potential to influence an organization’s behavior (Serinka et al., 2014). In the last two decades, the concept of organizational learning grew in academic publications as itself, and as a process of knowledge management (Patricia et al., 2013). The LO concept is often extended with three additional organizational principles: organizational learning, organizational knowledge, and management knowledge. Organizational learning is the process of transforming external market information into practical, contextual knowledge that informs practices across the organization (Crites et al., 2009). Recent emphasis in organizational achievement is put on using strategies which facilitate creating a “learning culture” in the organization. This new approach can help organizations to continue their improvements and to achieve their goal. Becoming a learning organization needs focusing on the future and on how to use previous accomplishments in order to improve. Some of the major characteristics of learning organization include: making a continuous improvement through internal processes, taking the best steps against competitors, providing correct and on-time education, consolidating managers’ positions as a key to improve performance via making use of training skills, providing opportunities for creativity in the working environment, using unexpected situations for learning, designing jobs appropriate to the skills of the staff and providing them with job satisfaction, trying to maintain staff’s commitment to achieve organizational goals, boosting staff’s commitment to share information and knowledge, and emphasizing joint decision. Like many of the organizational plans, the capability and probability of achieving the goal of becoming a learning organization depends on the manager’s value and commitment. To become learning organization, the most valuable and primary measure to be taken is a suitable and proper leadership in creating a supportive environment and taking the related steps (Hyslop, 2013). Because of this reason, organizations require faster, less costly, and more effective learning in the working environment. According to Marquardt learning can provide the best opportunity for not only the survival of organizations but also their success (Marquardt, 2006). Defined as an organization that facilitates learning of all its members, learning organization possesses certain characteristics to meet the ever-changing needs of the environment (Norashikin et al., 2014). A ‘‘learning organization is an organization skilled in creating, acquiring and transferring knowledge and at modifying its behavior to reflect new knowledge and insights’’. Thus, the organizational learning capability can be defined as the organizational and managerial characteristics that facilitate the organizational learning process or allow an organization to learn. From this perspective, the dimensions of the OL concept are its main facilitators within the organization (Barbaragón et al., 2014). In a learning organization there are lots of works to do for managers and to be a person who is unprejudiced, open to criticism, supporter of new ideas which is important for managers. Additionally, in order to increase learning capacity, managers should try to create environments in which the work done is questioned, the information and the experiences are being shared. At the learning organization, managers should be objective, open to new approaches, and keep themselves modern for adaptation and follow innovations. Managers should appreciate team-oriented studies to have a continuous learning environment. Furthermore they should always investigate and implement better systems to use at work. Great learning atmosphere must be established and organizations should encourage employees continuously (Serinkan et al., 2014).

World Health Organization considers learning in organizations as an effective and important factor to maintain the patients’ security (World Health Organization, 2009).

Moreover, not only can health systems as one of the most important social organizations promote learnability in their improvement process but they also can train their staff for participating in learning organizations (Yosefi et al., 2009). The significance of health systems in achieving the MDGs pertinent to health has received considerable attention in recent times (World Health Organization, 2009). On the other hand, health-care systems and organizations related to them have some specifically significant characteristics according to which they are of high importance. For example, doing various and vast activities, offering services of health, care, education, and research to the majority of people in the society, and playing the pivotal role in maintenance and development of health level are some of these fundamental characteristics. Also asserts that due to perceiving the significance of health as the main pivot of sustainable development, all of the relevant organizations should prioritize learning and permanent and purposeful education (Yosefi et al., 2009).

Due to the importance of the concept of organizational learning and health care organizations to become learning organizations, the present research aimed to assess LO profile of educational hospitals of Tehran and Qazvin Universities of Medical Sciences in Iran. To that end, the model of organizational interlocking systems, in line with the study conducted at Qazvin University of Medical Sciences in 2009, University was used.

## 2. Methodology

The present research was conducted using a cross-sectional method in the academic years of 2013-2014. Doctors, nurses, doctor assistants, non-medical staff (radiologist, physiotherapist, nutritionist, and laboratory expert), and administrative staff holding a B.S. and higher degrees participated in the study. They were selected from educational centers of Tehran University of medical sciences (prior to re-separation of Iran University of medical sciences) and Qazvin University of medical sciences. All of the eligible participants in the five educational hospitals of Qazvin province were subject to examination. Of the 25 educational hospitals in Tehran province, 9 of them were selected purposefully (based on specialty) and some of the eligible participants were investigated. Considering α=0.05 (95% confidence interval), β=0.2 (80% test magnitude), *σ*=30 and *d*=4 (2.5% relative error) and using the following formula, the sample size of 530 was determined:


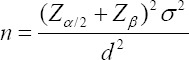


100 people (18.9%) of Qazvin educational centers and 430 people (81.1%) of Tehran educational centers took part in our study. The reason of our choosing is because of the number of Tehran’s hospitals. They are more than Qazvin’s hospitals.

Consistent with the instrument of LO profile, a questionnaire firstly designed by Marquart was used for data collection in the present research. It is a Likert-scale questionnaire involving 50 items. It assesses the 5 dimensions of LO, including “learning” dimension (items 1-10), “organization” dimension (items 11-20), “people” dimension (items 21-30), “knowledge” dimension (items 31-40), and “technology” dimension (items 41-50). In addition, the variables of age, gender, level of education, and years of service were included in the questionnaire.

The validity of the questionnaire was established by Mohebifar (Mohebbifar et al., 2012). Cronbach’s alpha coefficient was used to find out its internal consistency. In the current study, the Cronbach alpha coefficient was 0.98. To analyze the data, the performed statistical techniques were ANOVA, t-test, chi-square, correlation coefficients (Pearson and Spearman), and regression. Based on the findings of Berio’s (Berrio, 2006) study, the mean score of the 50 questions in the Likert-scale questionnaire was equal to 160, with the Standard Deviation of 27.

## 3. Result

530 questionnaires were distributed and collected. 90 of them were eliminated because of incomplete information provided by the respondents. Therefore, 440 (82%) questionnaires were completely responded. 449 (84.7%) respondents had a B.S. degree and 78 (15.3%) had an M.S. degree or higher. 360 of participants were females (67.9%) and 170 males (32.1%) (see Table 1.)

**Table 1:** Participants’ Demographic information

*Characteristics studied*	*Categories of* *Characteristics*	Frequency	*Percent*
**SEX**	Male	170	32.1
	Female	360	67.9
**AGE**	30≥	187	35.3
	31-40	153	28.9
	41≤	178	33.6
**EDUCATIONAL LEVEL**	DIPLOMA	6	1.1
	B.S	443	83.6
	M.S	78	14.7
	DOCTOR	3	0.6
**HISTORY**	≤5	182	34.3
	5.1-15	194	36.3
	≥15.1	115	21.7
**CITY**	Tehran	430	81.1
	Qazvin	100	18.9

The rating of learning organization profile in educational hospitals of Tehran and Qazvin universities of medical sciences was totally 123.6. Among the dimensions of learning organization, the lowest rating (22.63) was earned by “technology dimension” (a rating of 23.92 for men and 22.03 for women) and the “people dimension” gained the highest rating (25.71), with the rating of 25.11 for women and 26.98 for men. Based on level of education, the highest rating of learning organization was gained by those who had an M.S. degree or higher in the” people dimension” (29.69), and the lowest rating was obtained by B.S. holders in the “technology dimension” (22.05). In addition, there was a statistically significant difference between different levels of education for the dimensions of learning organization profile. In other words, higher level of education was associated with higher rating of LO profile.

The rating of learning organization profile in the hospitals of the two above-mentioned universities was a total of 123.76. Of the dimensions of learning organization, the lowest rating belonged to” technology” (22.63) and the highest rating to “people dimension”.

**Table 2:** Mean and standard Deviation of the rating of learning organization profile with its different dimensions

Descriptive Statistics					

	N	Minimum	Maximum	Mean	Std. Deviation
**Age**	518	22	67	36.66	9.148
**Work History**	491	.5	35.0	9.978	7.2636
**Learning**	502	10	50	25.35	8.043
**Organization**	488	10	50	25.40	8.327
**People**	505	10	50	25.71	8.361
**Knowledge**	500	10	50	25.37	8.121
**Technology**	504	9	45	22.63	7.695
**Total**	440	49	245	123.76	37.793

It was revealed that the mean of fivefold dimensions of learning organization was 121.21 and 129.11 for woman and men, respectively. Woman and men gained the highest rating in “people dimension” (25.11, 26.98). Also the lowest rating for both women and men was related to “technology dimension” (22.03, 23.92). Based on participants’ age, the highest rating belong to “people dimension” (25.70) and the lowest rating was related to dimensions of “learning”, “organization”, and “knowledge”, having the same rating of 25.34. It is necessary to mention that the statistical technique of regression was performed to identify the relationship between age and learning organization profile. The outcome revealed that it wasn’t statistically significant, P=0.025. In the dimensions of LO the difference between women and men wasn’t statistically significant, P=0.04. In determining learning organization profile in terms of level of education, it was revealed that the average rating of M.S./Ph.D. holders was 139.90 and it was 120.86 for B.S holders.

The highest rating of LO profile in terms of level of education was related to M.S./Ph.D. holders in “people dimension”(29.69) and the lowest rating belong to B.S. holders in “technology dimension” (22.05).

Additionally, there was a statistically significant difference between different levels of education in the dimensions of a LO profile, P=0.743, with higher level of education associated with higher rating of LO profile. In terms of years of service, the results revealed that the mean of five dimensions of LO for people of 5 years or less experience, 5.1-15 years experience, and 15.1 years or more experience was 121.194, 126.22, and 123.96, respectively. The highest rating was related to “people dimension” (25.87) and the lowest rating was related to “technology dimension” (22.72). It should be noted that there was not any significant correlation between LO profile and years of service, P=0.042.

The rating of LO profile in Qazvin province was 112.23 and in Tehran university of medical science was 126.56. The highest rating was gained by “people dimension” (26.44) in Tehran province and the lowest rating belonged to “technology dimension” in Qazvin province (19.90).

**Table 3:** Descriptive statistics for LO dimensions in Tehran and Qazvin

Group Statistics					

	CITY	N	Mean	Std. Deviation	Std. Error Mean
**Age**	**Tehran**	421	36.98	9.138	.445
	**Qazvin**	97	35.26	9.105	.924
**Work History**	**Tehran**	404	9.968	7.2202	.3592
	**Qazvin**	87	10.026	7.5045	.8046
**Learning**	**Tehran**	407	25.77	8.255	.409
	**Qazvin**	95	23.55	6.812	.699
**Organization**	**Tehran**	392	26.05	8.415	.425
	**Qazvin**	96	22.75	7.431	.758
**People**	**Tehran**	409	26.44	8.370	.414
	**Qazvin**	96	22.63	7.622	.778
**Knowledge**	**Tehran**	406	25.98	8.159	.405
	**Qazvin**	94	22.73	7.440	.767
**Technology**	**Tehran**	410	23.26	7.813	.386
	**Qazvin**	94	19.90	6.525	.673
**Total**	**Tehran**	354	126.56	38.356	2.039
	**Qazvin**	86	112.23	33.166	3.576

## 4. Discussion

Organizational learning is a dynamic process. Not only does learning occur over time and across levels, but it also creates a tension between assimilating new learning and exploiting or using what has already been learned. In today’s fast, competitive and innovative world, organizations must commit themselves to learn continuously. Human factor is the most important factor to create learning organizations. If people are diligent to improve themselves and given the opportunity to practice what has been learned to pass his career, it can be said that organizations have started to become a learning organization (Serinkan et al., 2014). Although there is a general recognition in the literature that training improves a firm’s performance, empirical research does not always provide evidence to support this effect. One possible explanation is that training does not have a direct effect on performance but an indirect effect by improving other organizational outcomes. The finding of Barb Aragón, Jiménez and Valle’s research suggests that organizational learning is one of those variables and that it mediates the relationship between training and performance and that the adoption of a learning-oriented training enhances performances through its positive effect on organizational learning (Barbaragón et al., 2014). World Health Organization (WHO) has codified some strategies by which we can effectively assist organizations to become a better learning organization. These strategies include: 1. Improving access to the world’s health information, 2. Translating knowledge into policy and action, 3. Sharing and reapplying experiential knowledge, 4. Leveraging e-Health in countries, 5. Fostering an enabling environment (World Health Organization, 2005).

Berio proposes a framework assessing the LO profile based on five subsystems: learning, organization, people, knowledge and technology. According to him, “learning” is placed in the center of the framework and other subsystems are derived from it. However, they are necessary for enhancing the quality and the effect of learning in the organization. In this study which was conducted in University of Ohio, the highest and the lowest ratings were related to “organization” and “technology”, respectively (Berrio, 2006).

In another research conducted by Mahmoodi, it was revealed that LO is capable of creating, acquiring transferring knowledge, and modifying its behavior reflects new knowledge and insights. With regard to the theory of job characteristics, he states that the staff is motivated to work if they feel their work is valuable and rewarding (Mahmoodi et al., 2014).

In a study, Tesai finds that there is a positive correlation between LO and internal marketing and organizational commitment. He asserts that internal marketing functions as a mediating factor between organizational learning and organizational commitment. Internal marketing helps hospital administrators to improve the quality of service provided by the staff and allows the organization to make a learning culture (Tsai, 2014). According to the findings obtained by Nodehi et al., creating knowledge in the organizations plays a key role in all learning organizations, and it is considered as a critical and important factor for success in the field of knowledge management. Their findings revealed that implementing the main elements of knowledge management in a LO and the world competition in communications are compulsory in an organization (Nodehi et al., 2013).

To become a LO and to institutionalize learning in an organization, not only the administrators but also the recognition of the beliefs of the people and their duties in the organization are of high importance. That is to say, adopting an approach related to the role of organization in knowledge management is very significant to become a LO (Mohebifar, 2007).

In line with the perspective of Islamic Republic of Iran in 1404 vision, when our country is developed and achieved the first place in terms of economy, technology and science in the region, and it is the Islamic and revolutionary identity inspiring in the world of Islam and constructive and effective in international relations, it necessitates our organizations to improve learning and become learning organizations (Habibi, 2009). In the present research, neither women nor men considered the hospital as a LO and in this regard, women showed the weaker profile. In contrast, in a study conducted by Berrio (Berrio, 2006), men had a weaker profile. Therefore, it can be deduced that the “gender” variable affects LO profile. Based on the findings gained by performing t-test, there was not any statistically significant difference between women and men and this is in line with the findings obtained in Berio’s (Berrio, 2006) study. It was also revealed that participants of different educational levels did not assess the hospitals as learning organization. In addition, participants of higher level of education visualized stronger profile of LO for hospitals. According to the present research, “the technology dimension” was the weakest dimension in hospital. Consequently, it asks for specific attention of stakeholders to move toward a LO. Norashikan, (Norashikin et al., 2014) maintains that the culture of learning organization has direct effects on organizational performance and organizational innovativeness, potentially leading to long-term organizational success. He defines LO as organization where people continually develop their capacity to achieve results they desire, whereby new patterns of thinking are nurtured, collective aspirations are freed and people learn to learn together.

A proper solution to create and institutionalize the “technology” dimension into the present organizations is the structure modification, modernization, and technology use in all levels of organization, leading to efficiency enhancement.

In the present research, after the “technology dimension”, the “learning dimension” was assessed as a weakest dimension. However, analyzing the five dimensions of organizational interlocking systems, Berio (Berrio, 2006) discovered that the “organization dimension” was the strongest dimension.

Habibi (Habibi, 2009) points out that it is necessary to launch a structural modification plan for obtaining superiority in learning to create organizational learning.

This change should involve adopting joint approach, fostering organizational learning culture, creating a strategy for building a LO and a structure to support the strategy, and developing a kind of leadership to acquire the highest motivation and performance. In addition, encouragement and support for team-oriented activities in conducting research and doing group projects, and creation of scientific-research networks can play influential roles in changing universities into learning organizations (Montazer rolfaraj et al., 2012).

In the present study, the “people dimension” was identified as the strongest dimension, but it was not considered as a feature of a LO from viewpoint of participants. Montazerolfaraj in his study identified two main dimensions, namely “individual sublimity” and “organizational sublimity” which individual sublimity takes priority over organizational sublimity (Montazerrolfaraj et al., 2012). Organizational learning is not always a linear process as stated by the model. Individual and group learning are parallel interacting and unfinished processes (Patricia et al., 2013). According to Bijani’s studies, knowledge management is one of the fundamental methods for organizations to become learning organizations. Knowledge management, knowledge sharing, and education of increasing organizational speed are effective in facilitating design and improvement processes and providing a positive working environment and reward system. These factors help the organization resist the changes and perform successfully (Bejany, 2009).

Keeping the above-mentioned points in mind, it should be noted that the structure and strategy of educational hospitals in Tehran and Qazvin have to change in various ways if they need to become learning organizations. By comparing the educational hospitals in Tehran and Qazvin, it was revealed that the rating of educational hospitals in Tehran was higher than that of educational hospitals in Qazvin. As a result, hospitals in Tehran were closer to the characteristics of a LO. In light of delivering various services including prevention, treatment, education, research and so on, the hospitals are required to take actions to become a LO.

Hence, health organizations have an appropriate background to be studied in the field of LOs; as a matter of fact, in addition to having a centered knowledge-based section, they provide specially complicated services, which are resulted from interaction of various sciences and require permanent modernization compatible with the latest technology and medical sciences (Satarighahfarokhi et al., 2012).

## 5. Conclusion

The results of the present study indicate that none of the selected hospitals in both provinces (Tehran and Qazvin) have the characteristics of a LO and they are far from it. Prior to their separation, Iran and Tehran universities of medical sciences had a better profile, comparing to Qazvin University of medical sciences. However, they are away from perfection. In fact, based on the findings, it can be concluded that educational centers in both universities perform poorly in five dimensions of LO. Therefore, these centers should exert all of their efforts to institutionalize learning in their organization.
